# Prospective Associations between Maternal Depressive Symptoms during Early Infancy and Growth Deficiency from Childhood to Adolescence

**DOI:** 10.3390/ijerph20237117

**Published:** 2023-11-27

**Authors:** Linda S. Pagani, Kianoush Harandian, Beatrice Necsa, Marie-Josée Harbec

**Affiliations:** 1 School of Psycho-Education, University of Montreal, Montreal, QC H3C 3J7, Canada; kianoush.harandian@umontreal.ca (K.H.); beatrice.necsa@umontreal.ca (B.N.); 2 School Environment Research Group, University of Montreal, Montreal, QC H3C 3J7, Canada; 3 Sainte-Justine’s Pediatric Hospital Research Center, University of Montreal, Montreal, QC H3T 1C5, Canada; 4 Institut National de Santé Publique du Québec, Montreal, QC H2P 1E2, Canada; marie-josee.harbec@inspq.qc.ca

**Keywords:** maternal depressive symptoms, child development, depression, child BMI

## Abstract

Maternal health represents an important predictor of child development; yet it often goes unnoticed during pediatric visits. Previous work suggests that mental state affects parenting. The relationship between infant exposure to maternal depressive symptoms suggests conflicting findings on physical growth. Body mass index (BMI) has not been rigorously examined across development. Using a prospective-longitudinal birth cohort of 2120 infants (50.7% boys), we estimated the prospective relationship between symptoms of maternal depressive symptoms at 5 months postpartum and later BMI in typically developing children. We hypothesized that maternal depressive symptom severity would predict later BMI through to adolescence. Mothers self-reported depressive symptoms at 5 months. Child BMI was measured by a trained research assistant at ages 6, 8, 10, 13, and 15 years. We estimated a series of sex-stratified regressions in which BMI was linearly regressed on maternal symptoms, while controlling for potential pre-existing/concurrent individual and family confounding factors. Boys born to mothers with more severe depressive symptoms at age 5 months had a significantly lower BMI than other boys at subsequent ages. There were no such associations observed for girls. Maternal depressive symptoms were prospectively associated with later BMI for sons and not daughters, predicting risk of faltering in growth through to adolescence. Health practitioners should routinely assess maternal psychological functioning during pediatric visits to optimize parent and child flourishment.

## 1. Prospective Associations between Maternal Depressive Symptoms during Early Infancy and Growth Deficiency from Childhood to Adolescence

Healthcare after childbirth is meant to ensure optimal growth and development for both mother and child [1]. Nevertheless, the focus is often centered on child well-being and maternal care falls through the cracks [2]. Yet, in most families, mothers remain charged with caregiving of new infants [3]. Consequently, the value of prioritizing maternal care to optimize subsequent child outcomes has been acknowledged by the World Health Organization [4].

The first year of infancy represents a transformative and challenging time for mothers [1,5]. There are neuroendocrine alterations, psychosocial adjustments, and the constant impact of infant-feeding and care [2]. It represents a critical period of vulnerability to changes in sleep patterns and feelings of sadness, anxiety, and social isolation [3]. The Center for Disease Control estimates that one in nine women meet diagnostic criteria for a psychiatric disorder in the first year postpartum [5]. Postpartum/postnatal (i.e., birth to approximately newborn age 2 months) and early childhood (i.e., from child age two months onward) depressive feelings and behavior in mothers represent the most common complication of childbearing [1]. Many newborns will therefore directly experience a certain degree of maternal functional impairment from negative emotion in their first year of life and beyond [1,6,7]. This incurs risks to both the affected women and their offspring in early childhood and beyond [5].

While social impairment is noted by family and friends, specific functional impairment risks for affected mothers in the first year may include a lack of interest in the baby and mother-child bonding, being overly preoccupied with being a bad mother or with the baby, and fear of harming the baby or oneself [7]. Children of mothers who are at risk of being less engaged in caregiving are more likely to experience less optimal physical and mental health outcomes [8]. Their intensity also predict risks for unhealthy body size and weight in infants [9,10,11,12].

Not much is known about the association between maternal depression during the infant bonding period and later indicators of body weight throughout childhood and adolescence. A review of nine prospective-longitudinal studies published prior to 2014, found evidence suggesting that chronic maternal depression might be associated with obesity risk [9]. However, this review combined diagnosis and symptomatology, the studies inconsistently implemented confound control, and not one was until adolescence. Within that review, one study found an inverse longitudinal relationship between maternal depressive symptoms at kindergarten and body mass index (BMI) in fifth grade [10]. Coinciding with that same time frame, a meta-analysis conducted in 11 low- and middle-income countries found that infant and toddler risk of being underweight was proportionately related to increases in maternal depressive symptoms [11]. This meta-analysis comprised many cross-sectional studies. A recent longitudinal study of mothers and their infants from the Infant Feeding Practices Study II found that higher early depressive symptoms at 2 months postpartum directly explained lower child BMI. They found that depressive symptoms in infancy affect feeding practices directly predicts lower BMI at age 6 years [12].

Growth faltering/failure refers to low length for age or low weight for length [13]. This conceptualization which reflects failure to thrive helps describe the relationship between maternal mental health and sub-optimal physical growth in children [14,15]. Although organic factors can predispose a child to have weight, height, and head circumference below standardized expectations, underlying medical grounds remain unidentified in more than 80% of atypically developing children [13]. Maternal difficulties may compromise infant nutritional intake [14]. Thus far, it remains unclear whether maternal depressive symptoms in infancy forecast long-term risks for growth in childhood and adolescence [15].

Past research on the association between maternal depressive symptoms and child physical development is characterized by several methodological flaws [2]. First, there is an over-representation of designs challenged by cross-sectional designs and selection bias, thus decreasing internal validity. Prospective methods offer control for pre-existing factors that could account for long-term observations [9]. Because longitudinal studies often do not control for child and family factors that might influence outcomes, they are not necessarily prospective. Second, sex has typically been treated as a nuisance variable that must be controlled. There are several exceptions [10,12,16]. Such an approach generates gender-neutral findings, yet daily life is confounded with gender expectations, limiting external validity [17,18]. Boys and girls experience risk and protective factors uniquely given differences in biological factors and sociocultural expectations regarding gender [19]. Early life exposure to family adversity, associated with depression and anxiety in mothers, has differential effects on males and females [16,20,21]. Boys, when compared with girls, are more generally vulnerable to the direct and indirect effects of maternal depression after childbirth and this is likely due to sex differences since conception [20,21]. It logically follows that the treatment of boys and girls as separate populations would enhance clinical generalizability of specific risk outcomes associated with maternal depressive symptoms [22]. The control variables act the same way, given that such variables also vary according to biological and social influences. Operationalization of this would be that girls with mothers with more depressive symptoms would be compared with girls with mothers with less depressive symptoms. The same should apply for boys of mothers with and without more depressive symptoms.

Using a longitudinal population-based birth cohort design, this study examines the association between maternal depressive symptoms in infancy and subsequent physical growth in typically developing children. Specifically, we aim to estimate the association between severity of depressive symptoms in mothers 5 months after childbirth and body mass index (BMI) in boys and girls from ages 6 to 15 years. We expect maternal depressive symptoms to be a risk factor for lower BMI, especially for boys.

## 2. Methods

### 2.1. Participants

**Figure 1:**
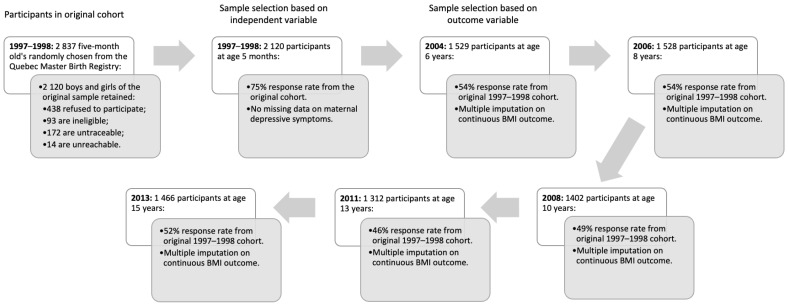
Flow chart of the participants from the Quebec Longitudinal Study of Child Development from 1997 to 2013.

The Quebec Longitudinal Study of Child Development (QLSCD) initially comprises 2837 children from the birth registry born between fall 1997 and 1998 in the province of Quebec, Canada. The sampling frame was divided into strata based on health and social service regions of the province. Stratification was employed to ensure representation of diverse regions within the province. Within each stratum, primary sampling units (PSU) were selected. These PSUs were equivalent to groups of local health and social service centers. Within each unit, secondary sampling units were selected such that they were representative of local health and social service centers. From this sampling frame meant to enhance the generalizability, a random sample of eligible children was selected for participation in this study. Data collection was planned and coordinated by the Institut de la Statistique du Québec (Montreal, QC, Canada). Of these: 93 children were ineligible because they were not provincial residents or had a first nation status; 172 were untraceable due to incorrect address information; 14 were unreachable (regardless of correct address); and 438 refused to participate. For our analysis, we used the remaining 2120 infants (50.7% boys) with parental consent that were deemed eligible for annual follow-up from age 5 months onward as our sub-sample. It represents 75% of the initial target population (see Figure 1). All participants had complete data on maternal depression at age 5 months and were thus included in this study. For this Institutional review board (IRB)-approved study, informed consent was obtained annually by parents in the early childhood phase, whereas both parents and children provided biennial consent during school-age years.

### 2.2. Measures

*Predictor Variable: Maternal Depressive Symptoms (Age 5 Months).* Mothers completed the abridged 13-item version of the Center for Epidemiologic Studies Depression Scale (CES-D), a clinically sensitive instrument that detects the clinical impairment threshold of a major depressive disorder [23]. Example items include poor appetite; could not shake off blues; felt depressed; everything seems effortful; restless sleep (α = 0.81 for male QLSCD infants and α = 0.78 for female QLSCD infants at 5 months) [23]. The French version of this established instrument has been found valid and reliable as a population-based screening tool in the assessment of severity of depressive symptomatology [24]. Frequency of symptoms were rated on a Likert scale: 1 = rarely or never (less than 1 day), 2 = some or a little of the time (1–2 days), 3 = occasionally or a moderate (3–4 days), and 4 = most or all of the time (5–7 days). Higher values are correlated with the depressive symptom severity [23].

*Outcome Variable: Body Mass Index (Ages 6, 8, 10, 13, and 15 years).* Using a standardized protocol, trained examiners measured child height and weight [25]. BMI was calculated based on the ratio of body weight in kilograms over height in meters squared. Scores were transformed into a standardized measure (*z*-scores), according to child sex [26].

*Control Variables: Potential Confounders in Early Childhood (Ages 5 and 17 Months).* Familial and child characteristics were considered to rule out pre-existing confounds and selection bias that could explain the link between maternal depression and failure to thrive in offspring. These were selected based on their substantive, theoretical, or established relationship with the predictor and outcome variables [1,2,3]. Given the link between negative feelings and lifetime antisocial behavior (comorbidity), we included maternal and paternal lifetime history of antisocial behavior using the National Institute of Mental Health-Diagnostic Interview Schedule 5 months postpartum (13 items and 12 items, respectively; 0 = below the median, 1 = above the median) [27]. Four indicators of adversity and financial hardship were included [2,28]: family configuration (0 = non-intact, 1 = intact); family income (0 = sufficient income, 1 = insufficient income; according to income-to-needs ratios for that year); family dysfunction (12 items; 0 = below the median, 1 = above the median); and maternal and paternal education (0 = completed high school, 1 = did not complete high school). Child neurocognitive skills at age 5 months were assessed through an adapted version of the Imitation Sorting Task, the “1, 2, 3 Hands Game”, which was administered by research assistants (0 = above the median, 1 = below the median) [29]. Using medical records obtained at age 5 months, preterm birth was established as born before 37 weeks of pregnancy (0 = not preterm, 1 = preterm) and weight for gestational age was estimated (g/weeks). Finally, fathers reported on difficult and unpredictable child temperament at age 17 months using the Infant Characteristics Questionnaire (ICQ; 10 items; 0 = below the median, 1 = above the median) [30].

### 2.3. Data Analytic Procedures

Using a sex-stratified approach to analyses, we estimated a series of least-square regressions in which BMI at ages 6, 8, 10, 13, and 15 years were linearly regressed on maternal depressive symptoms at age 5 months using SPSS v.26. The postulated relation can be interpreted as the prospective influence of increasing the severity of postpartum maternal depressive symptoms by 1 unit on the outcome of the ability to thrive, operationalized by BMI, at multiple time points (ages 6, 8, 10, 13, and 15 years). We document both unadjusted and adjusted models including pre-existing and concurrent child and family characteristics to reduce omitted variable bias in estimations of the association between maternal depressive symptoms and child BMI.

This study required follow-up data from several sources and waves. Figure 1 depicts a flow chart of the participants from the Quebec Longitudinal Study of Child Development from 1997 to 2013. These correspond to the predictors, outcomes, and potential confounders in early childhood. Attrition analyses were required to assess incomplete data as is the case in any longitudinal study. Using chi-squared tests, differences in individual and family characteristics in early childhood were found between participants with complete and incomplete data on outcomes variables. Girls and boys with incomplete data were more often born into families with insufficient income *X*^2^ (1, 1029) = 4.121, *p* < 0.05 and *X*^2^ (1, 1068) = 21.997, *p* < 0.001, respectively. Boys with incomplete data also had mothers with more antisocial antecedents *X*^2^ (1, 1043) = 8.410, *p* < 0.01 as well as mothers and fathers who had not completed high school *X*^2^ (1, 1081) = 15.060, *p* < 0.001 and *X*^2^ (1, 990) = 3.961, *p* < 0.05, respectively.

We used SPSS v.26 for multiple imputation to correct for response and attrition bias [31]. Using a stochastic algorithm, missing observations are imputed based on available complete data on auxiliary variables, creating multiple iterations generating variations in the original data. The algorithm generates slightly different values for each imputed measure across the multiple datasets. Our analyses were conducted with five imputed iterations [32,33]. The additional variance in response to differences in imputed values between the copies generated is added as a bootstrap to ensure the representativeness of the imputed values [31]. It is noteworthy that the analyses conducted on the original non-imputed subsample and the subsample corrected for response and attrition bias yielded similar results.

## 3. Results

### 3.1. Descriptive Analyses

Descriptive statistics for all study variables for boys and girls are reported in Table 1. Maternal depressive symptoms at age 5 months averaged 1.44 (standard deviation [SD] = 1.40) for boys and 1.37 (SD = 1.29) for girls, and 75% the sample had a score below 2.05. Roughly 10% of the sample has a score higher than 3. The average BMIs at ages 6, 8, 10, 13, and 15 years are reported in Table 1.

### 3.2. Baseline Characteristics as a Predictor of Severity of Maternal Depressive Symptoms in Infancy

Table 2 reports the adjusted unstandardized regression coefficients (with standard errors and 95% confidence intervals) reflecting associations between baseline child and family variables between ages 5 and 17 months and the severity of maternal symptoms at 5 months for boys and girls. For boys, insufficient family income (standardized b = 0.12, *p* ≤ 0.01), family dysfunction (standardized b = 0.31, *p* ≤ 0.001), and maternal antisocial antecedents (standardized b = 0.11, *p* ≤ 0.001) made unique significant contributions to depressive symptoms in mothers. For girls, in addition to family dysfunction (standardized b = 0.30, *p* ≤ 0.001), maternal antisocial antecedents (standardized b = 0.11, *p* ≤ 0.001) were also significantly associated with levels of depressive symptoms. 

### 3.3. Relationship between Baseline Characteristics and Subsequent Child BMI

Table 3 and Table 4, respectively, report the unadjusted and adjusted unstandardized regression coefficients for boys and girls. Preterm birth consistently associated with higher BMI from ages 6 to 15 years (standardized b_6years_ = 0.16 [*p* ≤ 0.001], standardized b_8years_ = 0.09 [*p* ≤ 0.05], standardized b_13years_ = 0.14 [*p* ≤ 0.001], and standardized b_15years_ = 0.10 [*p* ≤ 0.01] for boys and standardized b_8years_ = 0.08 [*p* ≤ 0.05], standardized b_10years_ = 0.13 [*p* ≤ 0.001], standardized b_13years_ = 0.12 [*p* ≤ 0.01], and standardized b_15years_ = 0.10 [*p* ≤ 0.01] for girls).

Higher weight for gestational age consistently associated with higher BMI from ages 6 to 15 years (standardized b_6years_ = 0.17 [*p* ≤ 0.001], standardized b_8years_ = 0.16 [*p* ≤ 0.001], standardized b_10years_ = 0.10 [*p* ≤ 0.05], standardized b_13years_ = 0.14 [*p* ≤ 0.001], and standardized b_15years_ = 0.13 [*p* ≤ 0.01] for boys and standardized b_6years_ = 0.18 [*p* ≤ 0.001], standardized b_8years_ = 0.14 [*p* ≤ 0.001], standardized b_10years_ = 0.22 [*p* ≤ 0.001], standardized b_13years_ = 0.17 [*p* ≤ 0.001], and standardized b_15years_ = 0.11 [*p* ≤ 0.01] for girls).

Boys and girls with fathers that had not graduated from high school also had higher BMI at different ages (standardized b_8years_ = 0.12 [*p* ≤ 0.01], standardized b_10years_ = 0.08 [*p* ≤ 0.05], and standardized b_13years_ = 0.16 [*p* ≤ 0.001] for boys and standardized b_6years_ = 0.10 [*p* ≤ 0.05], standardized b_8years_ = 0.18 [*p* ≤ 0.001], and standardized b_13years_ = 0.13 [*p* ≤ 0.01] for girls).

Finally, boys with a more problematic temperament had lower BMI scores at ages 8, 10, and 13 years (standardized b_8years_ = −0.12 [*p* ≤ 0.01], standardized b_10years_ = −0.09 [*p* ≤ 0.05], and standardized b_13years_ = −0.10 [*p* ≤ 0.01]).

### 3.4. Relationship between Maternal Depressive Symptoms in Infancy and Subsequent Child BMI

As, respectively, reported in Table 3 and Table 4, the unadjusted and adjusted unstandardized regression coefficients (with standard errors and 95% confidence intervals) reflect associations between maternal symptoms at age 5 months and subsequent BMI throughout childhood for boys and girls. For boys, depressive symptoms were significantly associated with a lower BMI at all ages above and beyond pre-existing and concurrent child and family characteristics. Unit increases in depressive symptoms predicted the following unit decreases in BMI: 13% (age 6 years, *p* ≤ 0.001), 11% (age 8 years, *p* ≤ 0.01), 8% (age 10 years, *p* ≤ 0.05), 10% (age 13 years, *p* ≤ 0.05), and 12% (age 15 years, *p* ≤ 0.01). For girls, depressive symptoms were not associated with BMI outcomes.

## 4. Discussion

Childbearing poses risks to maternal health [18]. The months following childbirth are busy and stressful for all parents [1]. Maternal sensitivity and responsiveness to infant cues forecast optimal growth and development in children [3]. Infants exposed to less such maternal characteristics risk thriving less well [7,8,12,21]. Significant depressive symptomatology might go undetected beyond the first several months after childbirth [5].

Although many typically developing mothers experience a range of negative emotions at an intensity that affects caregiving of infants and parenting, [2,3] most do not achieve the criteria for Major Depressive Disorder [7], which include sadness or depressed mood; lack of pleasure/interest; sleep disturbance (insomnia or hypersomnia); significant weight loss or gain; reduced energy; diminished concentration or indecisiveness; agitation or motor retardation; feelings of worthlessness or guilt; and recurrent thoughts of death or suicide [6]. These symptoms are often associated with significant distress that causes functional impairment in both social and occupational spheres [7]. Many mothers feel ashamed (correlated with worthlessness and inappropriate guilt) of the symptoms and may try to hide them for weeks or months prior to diagnosis, with some never being diagnosed [2,3]. Consequently, mothers may go untreated for a long period of time, thus experiencing a persistent negative attitude and general irritability, which can be overlooked because these are confounded with family dysfunction [5].

It is important to conduct population-based research using symptom-based and not diagnosis-based data because, as with most diagnostic categories, many psychiatric problems emerge at low levels and then begin to gradually increase in range and significantly influence impairment if not attended to when emerging symptoms go unnoticed by significant others and professionals [34]. We found that, for male children only, as maternal depressive symptoms in early infancy increased, the risk of having lower BMI proportionally increased from middle childhood to adolescence.

Research suggests that male infants are more negatively affected by early environmental stress, compared to female infants [35,36,37,38]. According to an evidence-based neurobiological model of development and psychopathogenesis, the differences lie in their brain maturity for gestational age at birth, affecting social and emotional functions in the earliest stages of development [35]. This is likely due to differences in sex hormones and differing rates of early right brain maturation processes, favoring females [20]. Neural circuitry targeted for stress regulation and socioemotional functioning develops more slowly in male than in female brains throughout the perinatal period. This delay might partly explain why boys might be more vulnerable to social stressors such as maternal depression [20]. Some suggest that increases in emotional reactivity responses to maternal stress risks are associated with decreases in self-comforting in male compared with female infants [21]. Others suggest that gender differences in self-regulation favoring females might make male infants more dependent and thus more vulnerable to nurturing and protection offered by their primary caregiver [22]. Biologically advantaged social interaction abilities may thus be a protective developmental asset for girls for regulating stress and tension and therefore palliate the severed relationship with their depressive mothers [12]. Increased vulnerability in young boys to long-term growth faltering compared to girls might also be explained by chromosomal sex differences [20].

There are noteworthy predictors of maternal depressive symptoms in the middle of the first year. For boys, factors such as financial hardship and poor communication and problem solving that characterize family dysfunction in the home environment and having a mother with a history of antisocial behavior in emerging adulthood are significant to predicted depressive symptoms in mothers 5 months after birth. Similarly, family dysfunction and maternal antisocial behavior was also strongly related to depressive symptoms in mothers of girls at baseline.

Our findings are not without limitations. Foremost, a non-experimental design does not establish causation. Second, the findings address only one aspect of growth. Third, in this study, we controlled for child temperament from the father’s point of view to remove the influence of having a difficult child. Although it strengthened our confound control by giving a more objective report on child characteristics, it did not eliminate negative maternal perceptions of child temperament or demandingness [27,28,29,30]. The findings do not elucidate whether they are largely from unresponsive parenting that became habitual or from long-term maternal irritability or sadness. Finally, obtaining significant relationships that indicate risk, our representative sample was predominantly white and middle-class and this is to be noted. This implies that coefficients would be bigger in an impoverished or high-risk population.

This study has some key strengths. Foremost is that the historical data from this study are not challenge by the pervasive and ubiquitous existence of today’s distracting technologies and social media. As such, it offers a more valid attempt to investigate the link between maternal depressive symptoms which today, given the pandemic of negative emotion in emerging adults and young adults, speaks very frankly about the risks of intolerance to such emotion when unidentified and untreated [39]. It makes the case that we need to pay attention to mothers and their symptoms several months after the postpartum/postnatal period, regardless of whether they achieve diagnosis or not. Past studies, plagued by cross-sectional and gender-neutral approaches, have considered the possibility that maternal emotional state might predict obesity [9]. Our prospective design controlled for socio-demographic, individual, and family factors that could have explained the findings. By isolating our predictor, maternal depressive symptoms, this approach constructs an equation that models how population-based natural variations affect subsequent development. For example, being a boy is a risk factor for difficult temperament in infants [20]. Difficult temperament, which is a neurologically driven emotional self-regulatory problem in infants, increases risk of postnatal depression in women [21]. Furthermore, sex-stratification allowed us to consider how this persistently affects the physical maturation of males and females as separate populations as both sexes are subjected to different influences from conception [19,20].

## 5. Conclusions

Because the focus during the first year is the infant, depressive symptoms in mothers can go overlooked or are hidden due to shame, and thus are undertreated. Difficulties regulating infant sleep–wake schedules, as well as crying and feeding, may prolong or complexify maternal depressive symptoms [34]. Health practitioners should routinely assess maternal psychological well-being during pediatric visits at any age to optimize parent and child well-being. Nevertheless, the mental health assessment of mothers during such visits remains a challenge. Functional impairment varies with symptomatology and phenotypically differs across women, making it hard to discern by health care professionals in general or pediatric practice. For some, even a narrow range of symptoms can impair social and occupational functioning [5]. This is important for several reasons. There is reasonable concern for morbidity and mortality, especially when symptoms persist past the first six months post-partum. Moreover, varying levels of key depressive symptoms are also likely to persist in one form or another up to more than a decade after childbirth [33]. Significant intensity in symptoms should be prioritized for psychiatric evaluation because of its associated risks related to care of children [18]. Counselling on maintaining optimism, prospective hope, and a growth perspective has been effective with at-risk mothers [40]. Such techniques emanating from positive psychological approaches diminish negative affect and should be universally implemented with all mothers in the year following childbirth. Even clinical presentations that do not meet diagnostic criteria merit appropriate intervention.

## Figures and Tables

**Table 1:** Descriptive statistics for study variables.

	Boys			Girls		
	M (SD)	Categorical Variables (%)	Range	M (SD)	Categorical Variables (%)	Range
Predictors (5 months)						
Maternal depressive symptoms	1.44 (1.40)	-	0.00–10.00	1.37 (1.29)	-	0.00–10.00
Outcomes (ages 6 to 15 years)						
Child BMI (age 6 years)	15.88 (1.52)	-	11.10–27.14	15.74 (1.78)	-	11.92–31.00
Child BMI (age 8 years)	16.70 (2.08)	-	9.69–29.45	16.72 (2.25)	-	9.88–29.27
Child BMI (age 10 years)	18.48 (2.68)	-	12.90–33.10	18.47 (2.84)	-	13.00–41.30
Child BMI (age 13 years)	20.85 (3.34)	-	13.70–40.60	21.05 (3.35)	-	14.20–46.00
Child BMI (age 15 years)	21.20 (3.39)	-	12.80–41.30	21.30 (3.48)	-	14.60–46.60
Control variables						
Maternal antisocial antecedents (age 5 months) 1 = above the median	-	25.8	-	-	25.0	-
Paternal antisocial antecedents (age 5 months) 1 = above the median	-	44.7	-	-	41.3	-
Family configuration (age 5 months) 1 = non-intact	-	18.4	-	-	19.6	-
Family income (age 5 months) 1 = insufficient	-	25.8	-	-	22.2	-
Family dysfunction (age 5 months) 1 = above the median	-	48.0	-	-	47.6	-
Maternal education (age 5 months) 1 = did not finish high school	-	15.9	-	-	16.1	-
Paternal education (age 5 months) 1 = did not finish high school	-	21.9	-	-	23.5	-
Neurocognitive abilities (age 5 months) 1 = below the median	-	52.1	-	-	51.9	-
Preterm birth (age 5 months) 1 = preterm (<37 weeks)	-	5.3	-	-	4.5	-
Weight for gestational age (age 5 months)	56.43 (8.58)	-	15.23–86.15	55.29 (8.03)	-	18.47–82.83
Temperament problems (age 17 months) 1 = above the median	-	48.3	-	-	47.3	-

M = mean; SD = standard deviation; BMI = body mass index. Analyses corrected for attrition bias. Data were compiled from the final master file of the Quebec Longitudinal Study of Child Development (1998–2013), ©Gouvernement du Québec, Institut de la statistique du Québec.

**Table 2:** Adjusted unstandardized regression coefficients (with standard errors and 95% confidence intervals) reflecting associations between baseline child and family characteristics between ages 5 and 17 months and the severity of maternal depressive symptoms at 5 months for boys and girls.

	Maternal Depressive Symptoms (Age 5 Months)	
	Boys	Girls
	*b* (SE) [95% CI]	*b* (SE) [95% CI]
Maternal antisocial antecedents (age 5 months)	0.13 (0.11) [−0.09; 0.35]	0.38 (0.11) *** [0.16; 0.59]
Paternal antisocial antecedents (age 5 months)	0.08 (0.10) [−0.11; 0.27]	0.15 (0.10) [−0.04; 0.34]
Family configuration (age 5 months)	0.08 (0.15) [−0.23; 0.38]	−0.16 (0.14) [−0.43; 0.12]
Family income (age 5 months)	0.41 (0.13) ** [0.15; 0.67]	0.23 (0.13) [−0.03; 0.49]
Family dysfunction (age 5 months)	0.80 (0.10) *** [0.61; 0.99]	0.73 (0.09) *** [0.55; 0.91]
Maternal education (age 5 months)	0.03 (0.15) [−0.27; 0.32]	−0.9 (0.15) [−0.38; 0.21]
Paternal education (age 5 months)	0.11 (0.12) [−0.13; 0.36]	0.13 (0.12) [−0.12; 0.37]
Neurocognitive abilities (age 5 months)	0.00 (0.09) [−0.19; 0.18]	−0.08 (0.09) [−0.09; 0.26]
Preterm birth (age 5 months)	−0.07 (0.25) [−0.57; 0.42]	0.09 (0.28) [−0.45; 0.64]
Weight for gestational age (age 5 months)	0.01 (0.01) [−0.01; 0.02]	0.00 (0.01) [−0.01; 0.01]
Temperament problems (age 17 months)	0.15 (0.09) [−0.04; 0.33]	0.00 (0.09) [−0.18; 0.17]
Adjusted R^2^	0.134 ***	0.115 ***

** *p* ≤ 0.01, *** *p* ≤ 0.001. *b* = beta coefficient; CI = confidence interval; SE = standard error. Analyses corrected for attrition bias. Data were compiled from the final master file of the Quebec Longitudinal Study of Child Development (1998–2013), ©Gouvernement du Québec, Institut de la statistique du Québec.

**Table 3:** Unadjusted unstandardized regression coefficients (with standard errors and 95% confidence intervals) reflecting singular prospective-longitudinal associations between maternal depressive symptoms at age 5 months and body mass index at ages 6, 8, 10, 13, and 15 years for boys and girls.

		BMI				
		Age 6 Years	Age 8 Years	Age 10 Years	Age 13 Years	Age 15 Years
		*b* (SE) [95% CI]	*b* (SE) [95% CI]	*b* (SE) [95% CI]	*b* (SE) [95% CI]	*b* (SE) [95% CI]
Boys	Maternal depressive symptoms (age 5 months)	−0.04 (0.02) * [−0.08; −0.001]	−0.02 (0.02) [−0.06; 0.03]	0.01 (0.02) [−0.03; 0.05]	−0.01 (0.02) [−0.05; 0.03]	−0.01 (0.02) [−0.06; 0.03]
	Maternal antisocial antecedents (age 5 months)	−0.02 (0.07) [−0.15; 0.12]	−0.01 (0.07) [−0.14; 0.13]	0.06 (0.07) [−0.08; 0.19]	0.10 (0.07) [−0.04; 0.23]	0.15 (0.07) * [0.01; 0.28]
	Paternal antisocial antecedents (age 5 months)	0.15 (0.06) * [0.02; 0.27]	0.07 (0.06) [−0.06; 0.19]	0.08 (0.07) [−0.05; 0.20]	−0.02 (0.06 [−0.14; 0.11]	−0.02 (0.06) [−0.14; 0.11]
	Family configuration (age 5 months)	0.16 (0.08) * [0.01; 0.31]	0.20 (0.08) ** [0.05; 0.35]	0.14 (0.08) [−0.01; 0.29]	0.17 (0.08) * [0.02; 0.32]	0.18 (0.08) * [0.03; 0.33]
	Family income (age 5 months)	0.27 (0.07) *** [0.14; 0.41]	0.21 (0.07) ** [0.08; 0.34]	0.30 (0.07) *** [0.17; 0.44]	0.22 (0.07) *** [0.08; 0.35]	0.31 (0.07) *** [0.17; 0.44]
	Family dysfunction (age 5 months)	−0.02 (0.06) [−0.14; 0.09]	0.14 (0.06) * [0.02; 0.25]	0.04 (0.06) [−0.07; 0.16]	−0.01 (0.06) [−0.13; 0.11]	0.01 (0.06) [−0.11; 0.12]
	Maternal education (age 5 months)	0.06 (0.08) [−0.10; 0.22]	−0.03 (0.08) [−0.19; 0.13]	0.13 (0.08) [−0.03; 0.29]	−0.07 (0.08) [−0.23; 0.09]	0.33 (0.08) *** [0.17; 0.49]
	Paternal education (age 5 months)	0.09 (0.07) [−0.06; 0.23]	0.26 (0.07) *** [0.11; 0.40]	0.26 (0.07) *** [0.12; 0.40]	0.35 (0.07) *** [0.21; 0.49]	0.23 (0.07) ** [0.09; 0.37]
	Neurocognitive abilities (age 5 months)	−0.01 (0.07) [−0.12; 0.14]	0.01 (0.07) [−0.14; 0.13]	0.02 (0.07) [−0.15; 0.12]	−0.04 (0.07) [−0.09; 0.17]	−0.06 (0.07) [−0.07; 0.19]
	Preterm birth (age 5 months)	0.50 (0.13) *** [0.24; 0.76]	0.08 (0.13) [−0.18; 0.34]	0.11 (0.13) [−0.15; 0.37]	0.48 (0.13) *** [0.22; 0.74]	0.46 (0.13) *** [0.20; 0.72]
	Weight for gestational age (age 5 months)	0.01 (0.00) ** [0.002; 0.02]	0.02 (0.00) *** [0.01; 0.02]	0.01 (0.00) ** [0.002; 0.02]	0.01 (0.00) ** [0.003; 0.02]	0.01 (0.00) ** [0.004; 0.02]
	Temperament problems (age 17 months)	−0.09 (0.07) [−0.23; 0.05]	−0.25 (0.07) *** [−0.39; −0.11]	−0.21 (0.07) ** [−0.35; −0.08]	−0.22 (0.07) *** [−0.36; −0.09]	0.01 (0.07) [−0.13; 0.15]
Girls	Maternal depressive symptoms (age 5 months)	−0.02 (0.02) [−0.07; 0.03]	0.04 (0.02) [−0.01; 0.08]	0.06 (0.02) ** [0.02; 0.11]	0.05 (0.02) * [0.000; 0.09]	0.03 (0.02) [−0.01; 0.08]
	Maternal antisocial antecedents (age 5 months)	0.09 (0.07) [−0.05; 0.23]	0.19 (0.07) ** [0.05; 0.33]	0.08 (0.07) [−0.06; 0.22]	0.19 (0.07) ** [0.05; 0.33]	0.14 (0.07) * [0.000; 0.28]
	Paternal antisocial antecedents (age 5 months)	0.06 (0.07) [−0.07; 0.19]	0.04 (0.07) [−0.09; 0.17]	0.11 (0.07) [−0.02; 0.24]	0.15 (0.07)* [0.02; 0.28]	0.08 (0.06) [−0.05; 0.21]
	Family configuration (age 5 months)	0.34 (0.08) *** [0.19; 0.49]	0.25 (0.08) *** [0.10; 0.40]	0.44 (0.08) *** [0.29; 0.59]	0.23 (0.08) ** [0.08; 0.38]	0.10 (0.08) [−0.06; 0.25]
	Family income (age 5 months)	−0.06 (0.07) [−0.20; 0.09]	0.14 (0.07) [−0.01; 0.28]	0.38 (0.07) *** [0.23; 0.52]	0.29 (0.07) *** [0.14; 0.43]	0.28 (0.07) *** [0.14; 0.42]
	Family dysfunction (age 5 months)	0.18 (0.06) ** [0.06; 0.30]	0.12 (0.06) * [0.001; 0.24]	0.19 (0.06) ** [0.07; 0.31]	0.13 (0.06) * [0.01; 0.25]	0.17 (0.06) ** [0.05; 0.29]
	Maternal education (age 5 months)	0.19 (0.08) * [0.02; 0.35]	0.10 (0.08) [−0.06; 0.27]	0.46 (0.08) *** [0.30; 0.62]	0.40 (0.08) *** [0.24; 0.56]	0.37 (0.08) *** [0.21; 0.53]
	Paternal education (age 5 months)	0.28 (0.08) *** [0.13; 0.42]	0.34 (0.07) *** [0.20; 0.48]	0.31 (0.07) *** [0.16; 0.45]	0.40 (0.07) *** [0.25; 0.54]	0.26 (0.07) *** [0.12; 0.40]
	Neurocognitive abilities (age 5 months)	0.16 (0.07) ** [−0.29; −0.03]	0.20 (0.07) ** [−0.33; −0.06]	0.14 (0.07) * [−0.27; −0.01]	0.02 (0.07) [−0.16; 0.11]	0.02 (0.07) [−0.15; 0.12]
	Preterm birth (age 5 months)	−0.14 (0.15) [−0.43; 0.15]	0.08 (0.15) [−0.21; 0.37]	0.40 (0.15) ** [0.11; 0.68]	0.24 (0.15) [−0.05; 0.52]	0.24 (0.15) * [0.05; 0.63]
	Weight for gestational age (age 5 months)	0.02 (0.00) *** [0.02; 0.03]	0.02 (0.00) *** [0.01; 0.02]	0.02 (0.00) *** [0.01; 0.02]	0.01 (0.00) *** [0.01; 0.02]	0.01 (0.00) [−0.001; 0.01]
	Temperament problems (age 17 months)	−0.01 (0.07) [−0.15; 0.13]	0.05 (0.07) [−0.09; 0.19]	0.08 (0.07) [−0.06; 0.22]	0.02 (0.07) [−0.12; 0.16]	−0.08 (0.07) [−0.21; 0.06]

* *p* ≤ 0.05, ** *p* ≤ 0.01, *** *p* ≤ 0.001. *b* = beta coefficient; BMI = body mass index; CI = confidence interval; SE = standard error. Analyses corrected for attrition bias. Data were compiled from the final master file of the Quebec Longitudinal Study of Child Development (1998–2013), ©Gouvernement du Québec, Institut de la statistique du Québec.

**Table 4:** Adjusted unstandardized regression coefficients (with standard errors and 95% confidence intervals) reflecting prospective-longitudinal associations between maternal depressive symptoms at age 5 months and body mass index at ages 6, 8, 10, 13, and 15 years for boys and girls.

		BMI				
		Age 6 Years	Age 8 Years	Age 10 Years	Age 13 Years	Age 15 Years
		*b* (SE) [95% CI]	*b* (SE) [95% CI]	*b* (SE) [95% CI]	*b* (SE) [95% CI]	*b* (SE) [95% CI]
Boys	Maternal depressive symptoms (age 5 months)	−0.11 (0.03) *** [−0.17; −0.04]	−0.09 (0.03) ** [−0.16; −0.03]	−0.07 (0.03) * [−0.13; 0.002]	−0.08 (0.03) * [−0.14; −0.01]	−0.09 (0.03) ** [−0.16; −0.03]
	Maternal antisocial antecedents (age 5 months)	−0.17 (0.09) [−0.35; 0.01]	−0.11 (0.10) [−0.29; 0.08]	−0.08 (0.10) [−0.27; 0.11]	0.00 (0.09) [−0.19; 0.19]	0.04 (0.09) [−0.14; 0.22]
	Paternal antisocial antecedents (age 5 months)	0.19 (0.08) * [0.04; 0.35]	0.13 (0.08) [−0.03; 0.29]	0.13 (0.08) [−0.04; 0.29]	0.02 (0.08) [−0.14; 0.18]	−0.02 (0.08) [−0.18; 0.13]
	Family configuration (age 5 months)	0.04 (0.13) [−0.21; 0.28]	0.03 (0.13) [−0.23; 0.28]	0.01 (0.13) [−0.26; 0.27]	−0.01 (0.13) [−0.27; 0.24]	0.11 (0.13) [−0.13; 0.36]
	Family income (age 5 months)	0.17 (0.11) [−0.05; 0.39]	0.21 (0.11) [−0.02; 0.43]	0.29 (0.12) ** [0.06; 0.51]	0.23 (0.11) * [0.01; 0.45]	0.28 (0.11) ** [0.07; 0.50]
	Family dysfunction (age 5 months)	0.00 (0.08) [−0.17; 0.16]	0.22 (0.09) ** [0.05; 0.39]	−0.01 (0.09) [−0.19; 0.16]	−0.01 (0.09) [−0.17; 0.16]	0.02 (0.08) [−0.14; 0.18]
	Maternal education (age 5 months)	0.04 (0.12) [−0.21; 0.28]	−0.25 (0.13) * [−0.50; −0.002]	−0.15 (0.13) [−0.40; 0.11]	−0.36 (0.13) ** [−0.61; −0.11]	0.13 (0.12) [−0.11; 0.37]
	Paternal education (age 5 months)	0.07 (0.10) [−0.13; 0.27]	0.31 (0.10) ** [0.11; 0.51]	0.22 (0.11) * [0.005; 0.43]	0.41 (0.10) *** [0.20; 0.61]	0.12 (0.10) [−0.08; 0.32]
	Neurocognitive abilities (age 5 months)	0.08 (0.08) [−0.23; 0.07]	0.03 (0.08) [−0.19; 0.12]	0.07 (0.08) [−0.23; 0.09]	−0.04 (0.08) [−0.12; 0.19]	−0.05 (0.08) [−0.10; 0.20]
	Preterm birth (age 5 months)	0.80 (0.21) *** [0.40; 1.21]	0.45 (0.21) * [0.03; 0.86]	0.40 (0.22) [−0.02; 0.83]	0.74 (0.21) *** [0.32; 1.15]	0.51 (0.21) ** [0.11; 0.91]
	Weight for gestational age (age 5 months)	0.02 (0.01) *** [0.01; 0.03]	0.02 (0.01) *** [0.01; 0.03]	0.01 (0.01) * [0.002; 0.02]	0.02 (0.01) *** [0.01; 0.03]	0.02 (0.01) ** [0.006; 0.03]
	Temperament problems (age 17 months)	−0.09 (0.08) [−0.25; 0.06]	−0.25 (0.08) ** [−0.41; −0.09]	−0.19 (0.08) * [−0.35; −0.03]	−0.19 (0.08) ** [−0.35; −0.04]	−0.01 (0.08) [−0.16; 0.14]
	Adjusted R^2^	0.052 ***	0.065 ***	0.030 ***	0.079 ***	0.031 ***
Girls	Maternal depressive symptoms (age 5 months)	−0.02 (0.04) [−0.09; 0.05]	0.01 (0.04) [−0.07; 0.07]	0.01 (0.04) [−0.06; 0.08]	0.03 (0.04) [−0.04; 0.10]	−0.01 (0.03) [−0.07; 0.06]
	Maternal antisocial antecedents (age 5 months)	0.10 (0.10) [−0.09; 0.29]	0.20 (0.10) * [0.003; 0.39]	0.07 (0.10) [−0.12; 0.26]	0.13 (0.10) [−0.06; 0.32]	0.08 (0.09) [−0.10; 0.27]
	Paternal antisocial antecedents (age 5 months)	−0.02 (0.08) [−0.19; 0.14]	−0.04 (0.09) [−0.21; 0.13]	0.13 (0.08) [−0.04; 0.29]	0.07 (0.08) [−0.10; 0.23]	0.07 (0.08) [−0.09; 0.23]
	Family configuration (age 5 months)	0.48 (0.12) *** [0.24; 0.72]	0.36 (0.13) * [0.01; 0.50]	0.33 (0.12) ** [0.09; 0.57]	−0.01 (0.12) [−0.25; 0.23]	−0.08 (0.12) [−0.31; 0.16]
	Family income (age 5 months)	−0.25 (0.12) * [−0.48; −0.02]	−0.05 (0.12) [−0.29; 0.18]	−0.08 (0.12) [−0.31; 0.15]	0.00 (0.12) [−0.23; 0.23]	0.13 (0.12) [−0.10; 0.36]
	Family dysfunction (age 5 months)	0.22 (0.08) ** [0.05; 0.38]	0.16 (0.09) [−0.01; 0.33]	0.17 (0.08) * [0.004; 0.33]	0.10 (0.08) [−0.07; 0.27]	0.14 (0.08) [−0.02; 0.30]
	Maternal education (age 5 months)	0.05 (0.13) [−0.21; 0.31]	−0.18 (0.13) [−0.44; 0.08]	0.12 (0.13) [−0.14; 0.38]	0.18 (0.13) [−0.08; 0.44]	0.16 (0.13) [−0.09; 0.41]
	Paternal education (age 5 months)	0.26 (0.11) * [0.05; 0.47]	0.46 (0.11) *** [0.24; 0.67]	0.16 (0.11) [−0.05; 0.38]	0.32 (0.11) ** [0.11; 0.53]	0.17 (0.11) [−0.04; 0.38]
	Neurocognitive abilities (age 5 months)	0.17 (0.08) * [−0.32; −0.01]	0.20 (0.08) ** [−0.35; −0.04]	0.12 (0.08) [−0.27; 0.04]	−0.01 (0.08) [−0.14; 0.17]	0.08 (0.08) [−0.23; 0.07]
	Preterm birth (age 5 months)	0.24 (0.24) [−0.24; 0.72]	0.50 (0.25) * [0.01; 0.98]	0.78 (0.24) *** [0.31; 1.26]	0.72 (0.25) ** [0.23; 1.20]	0.58 (0.24) ** [0.11; 1.05]
	Weight for gestational age (age 5 months)	0.03 (0.01) *** [0.01; 0.04]	0.02 (0.01) *** [0.01; 0.03]	0.03 (0.01) *** [0.02; 0.04]	0.02 (0.01) *** [0.01; 0.03]	0.01 (0.01) * [0.003; 0.02]
	Temperament problems (age 17 months)	−0.05 (0.08) [−0.20; 0.11]	0.00 (0.08) [−0.16; 0.15]	0.07 (0.08) [−0.08; 0.22]	0.01 (0.08) [−0.15; 0.16]	−0.14 (0.08) [−0.29; 0.01]
	Adjusted R^2^	0.075 ***	0.065 ***	0.073 ***	0.049 ***	0.029 **

* *p* ≤ 0.05, ** *p* ≤ 0.01, *** *p* ≤ 0.001. *b* = beta coefficient; BMI = body mass index; CI = confidence interval; SE = standard error. Analyses corrected for attrition bias. Data were compiled from the final master file of the Quebec Longitudinal Study of Child Development (1998–2013), ©Gouvernement du Québec, Institut de la statistique du Québec.

## Data Availability

Restrictions apply to the availability of these data. Data was obtained from the Institut de la statistique du Québec and are available with the permission of the Institut de la statistique du Québec.
